# 

**Published:** 1885-11

**Authors:** 


PAGE.

ORIGINAL COMMUNICATIONS.

Intubation of the Larynx, with History of Cases. F. E. Waxham, M. D. ... 401

Report of a Case of Laparotomy for Gun-shot Wound of the Small Intestine and Mesentery. A. V. Park, M. D. ... 412

County Provision for the Insane. James G. Kiernan, M. D. ... 424

EDITORIAL.

County Provision for the Insane ... 439

Chicago as a Medical Centre ... 440

"Words and Their Uses" ... 440

The Ninth International Medical Congress ... 441

PAGE

FOREIGN CORRESPONDENCE.

New Methods in the Treatment of Cholera. A. Lagorio, M. D. ... 442

SOCIETY REPORTS.

Transactions of the Chicago Society of Ophthalmology and Otology ... 449

Transactions of the Chicago Medical Society ... 456

Transactions of the Chicago Gynæcological Society ... 466

BOOK REVIEWS ... 478

NEWS ITEMS ... 482

OBITUARY ... 486

EXTRACTS AND ABSTRACTS ... 487

EACHANGES RECEIVED ... 495

